# Treatment of Basicervical Femoral Fracture With Retractable Talon Hip Compression Screw

**DOI:** 10.7759/cureus.20951

**Published:** 2022-01-05

**Authors:** Abdulrahim Dündar, Deniz Ipek, Sinan Zehir

**Affiliations:** 1 Department of Orthopedics and Traumatology, Hitit University, Erol Olçok Training and Research Hospital, Çorum, TUR

**Keywords:** harris hip score, tip-apex distance, cephalomedullary nail, retractable talon, basicervical femoral fractures

## Abstract

Background

Basicervical femoral fractures (BFFs) are rare and biomechanically unstable. The goal of this study was to evaluate the effectiveness of the Talon™ DistalFix™ (Orthopedic Designs North America, Inc., Tampa, FL, USA) nail for the treatment of BFFs with a novel design.

Methodology

In this retrospective study, 25 patients with BFFs were analyzed between January 2016 and March 2020 at our institute. All patients were treated with the Talon™ DistalFix™ nail. Patients over the age of 60 years with basicervical fractures caused by low-energy trauma were included in this study. For inclusion into the study, the minimum follow-up time had to be longer than six months. The postoperative radiographic bone union, operative time, tip-apex distance (TAD), sliding distance of the lag screw, quality of fracture reduction, and major complications such as cut-out, non-union of the fracture, femoral head collapse, and cut-through were recorded. The Harris Hip Score was used to evaluate hip function at the end of the follow-up period.

Results

The mean follow-up period was 22 months (range, 16-28 months), and the mean age was 77.8 years (range, 61-91 years). The average sliding distance of the lag screw was 5.7 mm (range, 0.2-13.1 mm). The mean TAD of immediate postoperative view was 20.8 mm (range, 18.7-23.7 mm), and the TAD was <25 mm in all cases. Radiographic union was confirmed in most cases, and the average time for radiographic union was 18.8 weeks (range, 12-25 weeks). Most fractures (90.5%) had healed with no postoperative mechanical complications (cut-out, cut-thorough, or lateral wall fracture) at the final follow-up, except for two patients. One of the two patients had no evidence of union at six months, and mild varus reduction was observed in the other patient who had shortening of >10 mm.

Conclusions

According to the clinical and radiological findings of this study, treatment with the Talon™ DistalFix™ nail showed satisfactory results. Hence, it can be a suitable option in the treatment of BFFs.

## Introduction

Basicervical femoral fractures (BFFs) are rare compared to other fractures of the hip [1,2]. BFF is an intermediate fracture between intracapsular and extracapsular hip fractures, accounting for 1.8% to 3.5% of all hip fractures [3]. These fractures are considered to be in the borderline category between intertrochanteric and femoral neck fractures due to their anatomical location [4]. Compared with intertrochanteric and femoral neck fractures, BFFs are associated with greater biomechanical instability and a higher rate of implant-related complications, which often lead to reoperation, prolonged hospital stay, increased mortality, and higher cost. The treatment of patients with basicervical fractures is very challenging [5]. Because BFFs are more unstable than intertrochanteric fractures, the implant used to treat these fractures should be similar to that of unstable intertrochanteric femoral fractures [6].

In general, cephalomedullary nail (CMN) and sliding hip screw (SHS) have been used widely for the treatment of intertrochanteric fractures. However, CMN nail is a safe implant for the treatment of unstable intertrochanteric femur fractures and can be a better option for basicervical fractures [7]. There is currently limited evidence regarding the optimal treatment of basicervical fractures. Therefore, the indication of CMN for BFFs remains controversial, which makes it difficult to ascertain the choice of treatment for these fractures.

In this study, we used the Talon™ DistalFix™ (Orthopedic Designs North America, Inc., Tampa, FL, USA) nail system. The Talon™ DistalFix™ is equipped with four retractable anchors instead of CMN with a single screw. These anchors are deployable and engage the cortical bone to improve rotational control and resistance to cutout and provide compression.

The aim of this study was to evaluate the clinical and radiological findings of BFFs treated using CMNs with a novel Talon™ DistalFix™ nail system.

## Materials and methods

In this retrospective study, we examined 25 patients diagnosed with BFFs and treated with the Talon™ DistalFix™ nail system between January 2016 and March 2020 in our institute. The study protocol was approved by the local Institutional Review Board, and informed consent was obtained from all patients. We evaluated the clinical and radiographic records of all patients.

The diagnostic criteria for BFFs were as follows: (1) two-part fractures at the base of the femoral neck; (2) the fracture line medial to the intertrochanteric line, and the distal fracture line located above the intertrochanteric line; and (3) an intact lesser trochanter. BFF was diagnosed by three orthopedic surgeons based on preoperative X-ray images. Patients aged 60 years or more, being treated with the Talon™ DistalFix™, having low-energy trauma, and a follow-up duration exceeding six months were included in this study. Patients with high-energy trauma, fractures irreducible by closed methods, history of hip fractures, surgical treatment with open reduction, pathological trauma and malignancy, transcervical fractures, and the lesser trochanter with separated fragment were excluded from the study.

The patients were evaluated on follow-up visits at six weeks, three months, six months, one year, and two years. The clinical and radiographic assessment, the progress of fracture healing, and complications were assessed at regular intervals. Data such as age, sex, American Society of Anesthesiologists (ASA) score, operative time, tip-apex distance (TAD), the sliding distance of the lag screw, and the quality of fracture reduction were recorded. In addition, we recorded major complications such as cut-out, non-union of the fracture, femoral head collapse, and screw protrusion. The Harris Hip Score was used to evaluate the hip function at the end of the follow-up period, and the results were categorized as excellent (90-100), good (80-89), fair (70-79), or poor (<69). The quality of fracture reduction was evaluated based on the classification provided by Fogagnolo et al. [8] using postoperative X-ray images, which were classified as good, acceptable, or poor. The TAD was measured using the anteroposterior and lateral X-rays [9]. The minimum follow-up period was at least six months. All implants were inserted by three experienced surgeons using the same standard technique.

Surgical technique

**Figure 1:**
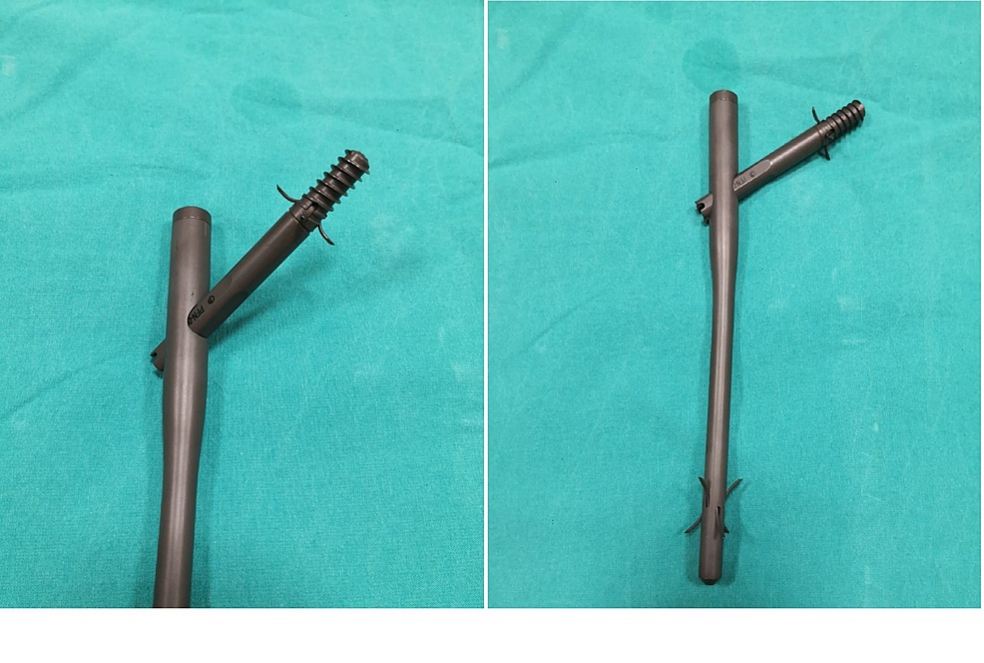
The Talon™ DistalFix™ nail. Four retractable anchors and the lag screw of the proximal femoral nail.

All procedures were performed on a traction table in the supine position under general or regional anesthesia. Closed reduction was performed and confirmed using the C-arm image intensifier. The reduction status was confirmed with anteroposterior and lateral views of fluoroscopic images. When the reduction status was good or acceptable on both views, a Talon™ DistalFix™ nail was inserted into the distal femoral fragment (Figure 1). Then, a Talon nail was deployed and six retractable anchors were penetrated into the femoral cortex, which provided axial and rotational stability for distal locking. After insertion of the Talon nail, the guide pin was inserted slightly inferior to the neck center on the anteroposterior view and centered on the lateral view. The lag screw was placed over the guide pin and the screw was fully deployed, which resulted in the four retractable anchors penetrating into the dense bone strongly.

After the day of operation, passive range of motion exercises were performed for the knee and hip, and sitting was allowed. Partial weight-bearing with a walker was allowed approximately three weeks after the surgery as tolerated by the patient, while full weight-bearing was allowed by the eighth postoperative week depending on systemic condition and pain.

Statistical analysis

SPSS version 23.0 (IBM Corp., Armonk, NY, USA) was used for statistical analysis. Descriptive statistical analyses were performed to describe the patient population and surgical procedure.

## Results

In this study, we examined 25 (3%) patients diagnosed with BFFs among the 830 patients treated for hip fractures between January 2016 and March 2020 in our institute. During the follow-up period, three patients were lost to follow-up, and one patient died less than six months after the injury and before the bony union. Overall, 21 (nine male and 12 female) patients with a mean age of 77.8 years (range, 61-91 years) were included in this study. Demographic and operative data of patients are shown in Table 1.

**Table 1:** Demographic and operative data of the patients. Values are presented as number, mean (range ), or number (%). ASA: American Society of Anesthesiologists

Variable	Data
Number of patients	21
Age (years)	77.88 (61–91)
Side	
Right	10
Left	11
Operation time (minutes)	35.48 (28–48)
Gender	
Male	9
Female	12
ASA score	3.05 (2–4)
Follow-up time (months)	22.27 (16–28)

The average sliding distance of the lag screw was 5.7 mm (range, 0.2-13.1 mm). The mean follow-up period was 22 months (range, 16-28 months). TAD was measured on the postoperative anteroposterior and lateral X-ray images. The mean TAD of immediate postoperative view was 20.8 mm (range, 18.7-23.7 mm), and TAD was <25 mm in all cases. Radiographic healing was confirmed in all cases, and the average duration for the radiographic union was 4.6 months (range, 3-8 months). Most fractures (90.5%) healed with no postoperative mechanical complications during the final follow-up visit, except for two patients. One of the two patients had no evidence of union at six months and underwent arthroplasty. The patient was placed in the lateral decubitus position on the other side and the end-cup, lag screw, and distal nail talon were respectively removed. The implant was removed without any difficulty and arthroplasty was successful. Mild varus reduction was observed in one patient who had shortening of >10 mm (Table 2). The reduction quality was assessed using immediate postoperative radiographs. Overall, 18 (85.7%) patients were classified as good, two (9.5%) as acceptable, and one (4.8%) as poor (Table 2). The average operative time was 35.4 minutes (range, 28-48 minutes), with no intraoperative complications. The complication rate in the current study was 9.5% (2/21). The reduction quality of one of the 21 patients was poor with no evidence of union at six months, and one patient had shortening of >10 mm. There was no evidence of fixation failure of the femoral head at the end of the postoperative follow-up period. The Harris Hip Score was excellent in 14 patients, good in six, and fair in one (Table 2). Two patients had slight persistent pain possibly due to prominent implant; however, these patients were able to walk with a crutch and refused implant removal. Four patients experienced postoperative medical complications such as pneumonia, delirium, and urinary tract infection.

**Table 2:** Clinical and radiological outcomes of patients with basicervical femoral fractures. Values are presented as number (%), mean (range), or mean ± standard deviation.

Radiologic parameter	Data
Tip-apex distance (mm)	20.81 (18.7–23.7)
Sliding distance of lag screw (mm)	5.71 (0.2–13.1)
Shortening of >10 mm	1 (4.8)
Screw protrusion	0
Screw cut-out	0
Non-union	1 (4.8)
Time for radiographic union (months)	4.6 (3–8)
Quality of fracture reduction	
Good	18 (85.7)
Acceptable	2 (9.5)
Poor	1 (4.8)
Harris Hip Score	88 ± 3.1
Excellent	14
Good	6
Fair	1
Poor	0

**Figure 2:**
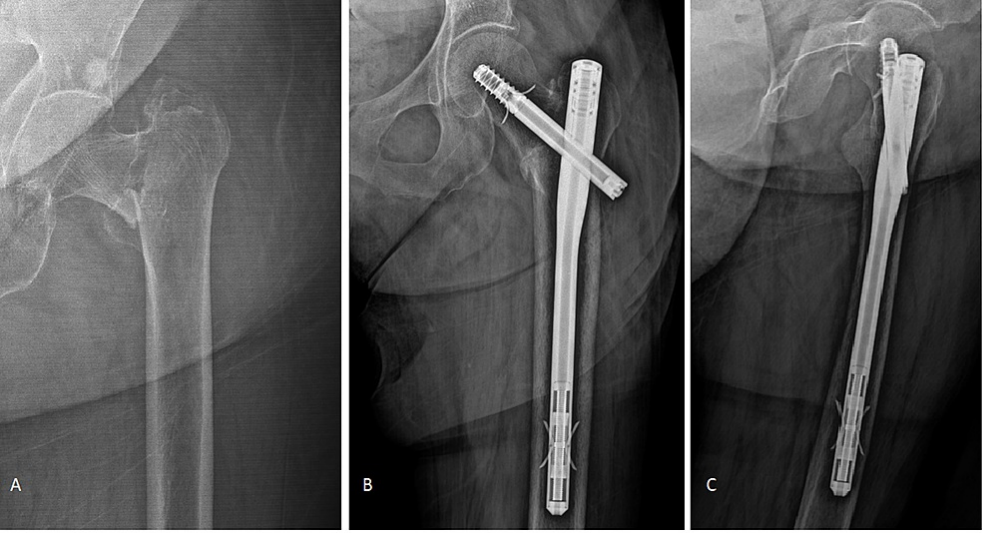
(A) Radiograph of a two-part basicervical fracture in an 86-year-old man. (B, C) Postoperative anteroposterior and lateral radiographs before completion of follow-up.

**Figure 3:**
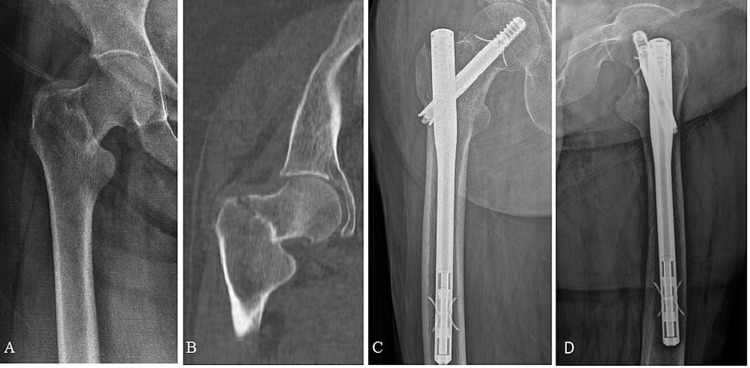
(A) Radiograph of a two-part basicervical fracture in a 91-year-old female. (B) A computed tomography image shows the coronal section of the fracture level. (C, D) Postoperative anteroposterior and lateral images at six months show no telescoping.

Figure 2 shows the radiographs of an 86-year-old male, and Figure 3 shows the radiographs of a 91-year-old female. Both patients had a two-part basicervical fracture.

## Discussion

BFFs have not been well identified in existing classification systems and are relatively rare. High failure rates have been reported for these fractures when treated with osteosynthesis [10]. Intramedullary and extramedullary fixation are the two primary treatment options for two-part BFFs [11]. Several studies have emphasized that the use of SHS is challenging to provide stable fixation in BFFs and cephalomedullary nailing may provide better clinical outcomes [12]. In our department, instead of SHS and multiple screw fixation, CMNs are always used to treat unstable trochanteric fractures such as BFFs due to the challenging management of these fractures. Su et al. showed that BFFs had higher biomechanical instability than other proximal femoral fractures and recommended that BFFs should be treated as unstable trochanteric fractures [13]. The prevalence of BFFs varies from 1.8% to 3.5% [1,3]. Our results showed that the prevalence of BFFs was 3% (25/830), which was consistent with the prevalence reported by previous studies. Various fixation devices have been used for the treatment of BFFs. However, the definition and optimal treatment of BFFs remain controversial [14]. CMN is more efficient in the treatment of BFFs and allows early exercise; however, Bojan et al. [10] showed a comparatively high incidence of screw cut-out in BFF treated by CMN [15]. Watson et al. [15] also reported that intramedullary fixation was not efficient in the treatment of BFF, with a high failure rate (54.5%) [16]. Some studies have reported good results of CMN for the treatment of BFFs [17], while others have reported a higher treatment failure rate [10]. However, according to the clinical and radiographic findings of our study, the Talon™ DistalFix™ provided good fracture union and a lower incidence of fixation failure. Most fractures (90.5%) healed with no postoperative mechanical complications, and the complication rate was 9.5% (2/21) in this study, which was different from the rate reported by Watson et al. In our view, the high failure rate of their study was most probably caused by an implant-related problem of the nail they used or mechanical application.

The Talon™ DistalFix™ can be an appropriate choice for the treatment of unstable intertrochanteric fractures [18]. We observed good results in unstable intertrochanteric fractures such as BFFs treated with Talon nails. Zehir et al. compared three different CMN systems for the treatment of unstable intertrochanteric hip fractures and reported that the Talon™ DistalFix™ was associated with lower cut-out rates than proximal femoral nail antirotation [19]. A patient with a TAD of >25 mm most probably has cut-out [20]. In the present study, the mean TAD, defined as the prediction of screw cut-out, was 20.8 mm (range, 18.7-23.7 mm), and TAD was <25 mm in all cases, with satisfactory TAD and Harris Hip Scores. All lag screws were inserted slightly inferior to the neck center on the anteroposterior view and centered on the lateral view, which resulted in the four retractable anchors penetrating into the dense bone strongly as well as stable fixation and improved fracture healing (Figure 3). A previous biomechanical study reported that Talon deployment significantly enhanced interfragmentary compression and rotational strength. Inserting the lag screw in the inferior position is the crucial technical step to optimize the Talon lag screw purchase [21]. We also inserted the lag screw slightly inferior to the neck center on the anteroposterior view and centered on the lateral view.

Talon™ DistalFix™ devices provide an innovative technique for distal and proximal femoral fixation, which feature eliminates the requirement for distal locking screws, complications associated with locking screw placement, additional surgical incision, and longer operative time. All of these can decrease morbidity, infections, blood loss, and anesthetic complications. Given the rarity of this type of fracture, the present study adds more evidence regarding the efficacy of fixation for this special type of fracture using the Talon™ DistalFix™ nail.

This study has some limitations. First, this was a retrospective study. Second, this study had a limited sample size owing to our rigorous inclusion criteria and the rarity of basicervical hip fractures. Further prospective randomized studies comparing Talon and other devices are needed to verify the appropriate treatment for BFFs.

## Conclusions

This retrospective study showed a good treatment outcome with the Talon deployment nail system for strictly defined two-part BFFs. However, larger sample sizes and longer follow-up periods are needed to confirm the optimal treatment method for these specific fracture patterns. We suggest that the Talon™ DistalFix™ may be a suitable option for BFFs.
